# Latest trends in *L. infantum* infection in dogs in Spain, Part II: current clinical management and control according to a national survey of veterinary practitioners

**DOI:** 10.1186/s13071-020-04080-8

**Published:** 2020-04-21

**Authors:** Ana Montoya, Rosa Gálvez, Rocío Checa, Juliana Sarquis, Alexandra Plaza, Juan Pedro Barrera, Valentina Marino, Guadalupe Miró

**Affiliations:** Grupo de investigación Epicontrol-Carnívoros, Departamento de Sanidad Animal, Facultad de Veterinaria, Universidad Complutense de Madrid, Madrid, Spain

**Keywords:** *Leishmania infantum*, Survey, Questionnaire, Clinical management, Canine leishmaniosis, Feline leishmaniosis, Spain

## Abstract

**Background:**

Canine leishmaniosis (CanL) is a parasitic zoonotic disease, endemic in the Mediterranean basin including Spain. While knowledge about CanL, its management, treatment, prevention and control mounts, it remains unclear whether all clinical veterinarians follow the same international recommendations, such as those of the LeishVet group. This study was thus designed to assess recent trends in the clinical management of CanL in veterinary clinics across Spain through a questionnaire-based survey. Results were compared with those of a prior national multicenter questionnaire administered by our research team in 2005.

**Methods:**

A questionnaire consisting of 28 questions about CanL was developed using Google Forms and distributed by email to 1428 veterinary clinics in Spain. Questions were designed to obtain data on common clinical signs, techniques and complementary exams used to diagnose the disease, and on its monitoring, treatment and control measures. Data were collected in a database for statistical analysis.

**Results:**

Completed questionnaires were returned by 295 clinics. Compared to the situation in 2005, responses indicate that clinical signs of CanL have not changed significantly, cutaneous lesions being still the most prevalent sign observed by practitioners. Quantitative serological techniques are considered an adequate approach to diagnosis, provided their results are supported by the findings of a thorough physical exam, as well as complementary tests (complete blood count, biochemical profile, plasma protein electrophoretogram and complete urinalysis). Treatment protocols and check-ups follow international recommendations. Finally, a multimodal approach is being endorsed to adequately control CanL including preventive measures such as annual serological check-ups and the combination of repellents and vaccines. Additionally, owners are being better informed about CanL by veterinarians, which translates to the improved control of this zoonosis.

**Conclusions:**

The clinical management of CanL has recently undergone significant changes owing to improvements in clinical knowledge of the disease, more unified international criteria, improved diagnostic techniques and their adequate interpretation, as well as a greater awareness of the disease transmitted to owners.
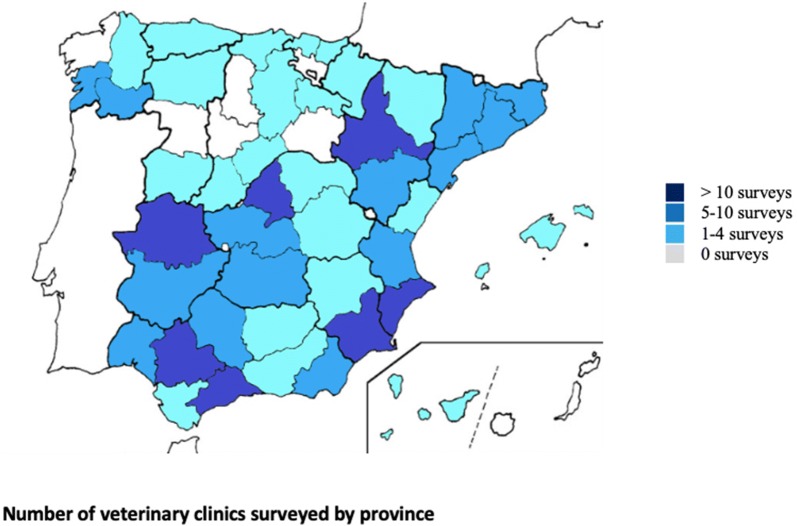

## Background

Canine leishmaniosis (CanL) is an important parasitic zoonotic disease caused by *Leishmania infantum*, endemic in the Mediterranean basin including Spain. The disease is transmitted by female blood-feeding phlebotomine sand flies, and dogs (*Canis familiaris*) are both its natural host and the major reservoir of infection for humans and other animals [1, 2]. The presence of *L. infantum* in felids and other animals has also been confirmed, although so far there are few data on these species despite being considered a potential secondary reservoir for the infection of humans and other animals [3–7]. The closest example is the largest outbreak of human leishmaniasis known in Europe occurring in 2009 in the southwest of Madrid [8] in which hares (*Lepus granatensis*) and rabbits (*Oryctolagus cuniculus*) were identified in xenodiagnostic and molecular diagnostic studies as reservoirs and held responsible for the transmission of human leishmaniosis [9, 10]. The epidemiological role of wildlife species has not yet been established [4, 11–13] yet several authors propose that these animals can act as sentinels as they indicate the risk of transmission to other animals (domestic and wild) or even to humans, highlighting the importance of the concept of “One Health” for the control of leishmaniosis [14–16].

In Spain, the seroprevalence of CanL differs from one area to another, depending on environmental factors such as temperature, humidity, geographical location, density and dispersion of the vector [1, 17]. In cats, seroprevalence rates provided in numerous studies have not been negligible, yet significantly lower than those observed in the canine population [18].

The most important characteristic of CanL is its extraordinary clinical polymorphism. This determines that a thorough assessment including medical history and physical examination are mandatory to confirm a causal relationship between *Leishmania* infection and the clinical signs presented by the animal. The diagnostic techniques used are based on the detection of the parasite (cytology, culture, molecular techniques, etc.) and anti-*L. infantum* antibodies (serological techniques). However, complementary diagnostic tests such as blood tests (blood count and biochemical profile), urine tests (e.g. urinalysis, urine protein/creatinine ratio (UPC)), ultrasound, etc. are required to identify the clinicopathological abnormalities associated with the disease and thus assess the general disease status of the animal and monitor its clinical progression after treatment [2, 19, 20].

With regard to the treatment of CanL, progress has been limited. Although treatment and clinical follow-up protocols have markedly advanced, there is currently no treatment capable of parasitological cure or of avoiding relapse. Prevention is the best way to fight the disease, greatly helping to stop the spread of infection to other animals and humans. However, at present, no preventive measure offers 100% guarantee. Thus, recommendations are adequate control of the vector, early diagnosis, and the treatment of sick dogs according to their clinical stage depending on their clinical signs [2, 21, 22]. The development and appearance of new vaccines for CanL is a new strategy for the control of this important zoonosis [21, 23, 24].

Knowledge about CanL, its management, treatment, prevention and control is on the increase. However, it is unknown whether all clinical veterinarians follow the same international recommendations such as those of the LeishVet group [2, 21]. Consequently, the aim of the present study was determine how CanL is clinically managed *via* a multicentre questionnaire completed by veterinarians throughout Spain. Results were then compared with those of a similar national multicentre questionnaire developed by our research team in 2005 [25].

This study is Part II of a larger investigation addressing the current situation of CanL is Spain. In Part I, we mapped seroprevalences of infection in dogs across the country based on reported and our own more recent data, and also provided sand fly species distributions and addressed factors affecting their distribution and density.

## Methods

### Questionnaire

The questionnaire (Additional file 1: Text S1), consisting of 28 questions about CanL clinical management, was developed through Google Forms. The items included were the same as in a previous national multicenter questionnaire developed by our research team in 2005 [25]. In this questionnaire, information is obtained about the characteristics of the veterinary clinics, the incidence and prevalence of *L. infantum* infection, the clinical signs observed, the diagnostic techniques and the complementary analyses used for the diagnosis of CanL and its monitoring, treatment, disease progression, control measures, vaccination and information provided to the owner.

Responses were anonymous and it was assured by collecting email addresses that questionnaires were not completed by more than one veterinarian at the same veterinary clinic. Email addresses were obtained through the Association of Spanish Veterinarian Specialists in Small Animals (AVEPA), the Veterinary Colleges of the different provinces and in a web search.

### Statistical analysis

All data were collected in a database (Microsoft Excel 2010) for statistical analysis. In addition to descriptive statistics of the survey responses, we analysed the data in an effort to understand possible associations between the incidence and progression of *L. infantum* infection and geographical area. Differences were also explored in the responses regarding diagnostic techniques, management of leishmaniosis (treatment and follow-up) and prophylactic measures provided by veterinarians who indicated they used LeishVet guidelines *versus* those who did not. For this purpose, we used the Chi-square test (SPSS 21.0). Significance was set at *P* ≤ 0.05.

## Results

### General characteristics

**Fig. 1:**
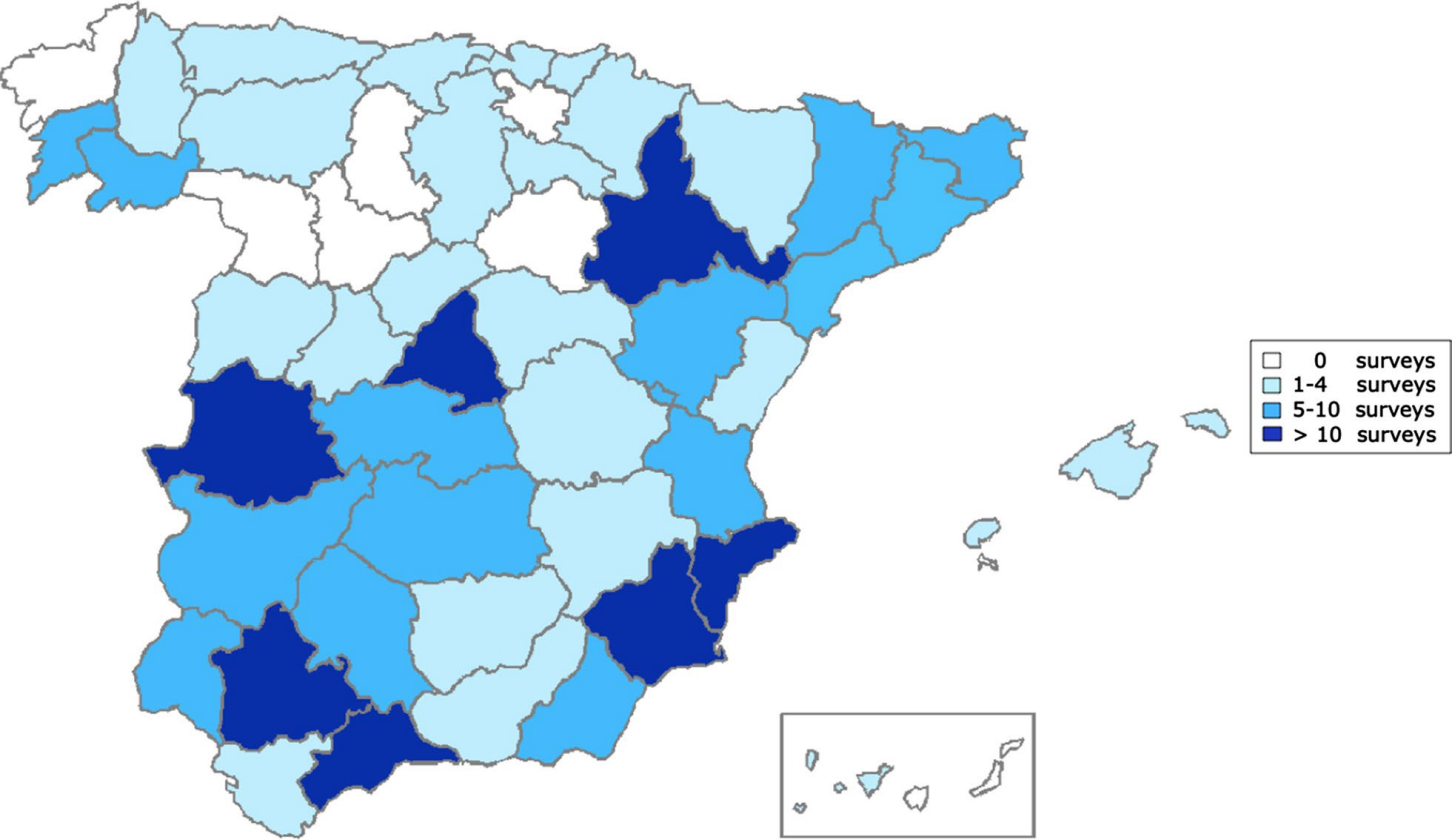
Number of veterinary clinics surveyed by province

The questionnaire sent by email to 1428 veterinary clinics in Spain was completed by 295 clinics/veterinarians. The geographical distribution of these clinics covering 43 of the 50 Spanish provinces are shown in Fig. 1. According to the CanL infection risk based on seroprevalences recorded in Spain) (the number of veterinary clinics surveyed (*n*) is provided alongside seroprevalence) were: Canary Islands (*n *= 1; 0.4%), northern Spain (*n *= 32; 11.6%), central Spain (*n *= 114; 41.5%) and south and east Spain (*n *= 128; 46.5%). In most veterinary clinics, the average number of veterinarians was 1–4 (81.7%), and 64.4% examined more than 10 animals per day.

The different canine vector borne diseases (CVBD) diagnosed by veterinarians were leishmaniosis (92.5%; 273/295), ehrlichiosis (48.8%; 144/295), anaplasmosis (20.7%; 61/295), dirofilariosis (15.6%; 46/295), piroplasmosis (9.5%; 28/295), borreliosis (1%; 3/295) and ricketsiosis (0.7%; 2/295). The presence of some CVBD was associated with geographical distribution such as dirofilariosis (South of Spain, East of Spain and Canary Islands) and piroplasmosis (North of Spain) while leishmaniosis, ehrlichiosis and anaplasmosis showed a homogenous distribution pattern across Spain. Borreliosis and ricketsiosis were not assessed due to insufficient data.

With regard to the incidence of *L. infantum* infection, the perception of 50.2% of veterinarians was that it remains stable, while 34.9% believe its incidence has increased and 14.9% it has decreased. Significant differences were also observed (*χ*^2^ = 23.257, *df* = 6, *P* = 0.001) in the perceptions of veterinarians in different geographical areas: 65.6% of the veterinarians working in northern Spain thought that CanL is increasing in incidence while in other areas, veterinarians (49.2–57%) felt that CanL infection has stabilized.

Few data about feline leishmaniosis (FelL) were reported. Twenty-four of 295 (8.1%) participants indicated that they had diagnosed at least one cat infected with *L. infantum*. No significant differences in FelL were observed with respect to the geographical area (*χ*^2^ = 1.050, *df* = 2, *P* = 0.592).

### Clinical signs

**Fig. 2:**
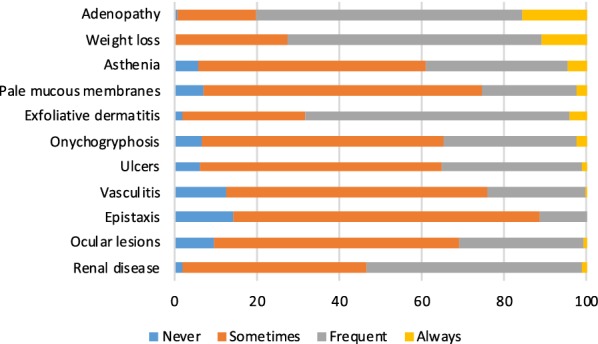
Clinical signs of CanL observed by practitioners and their frequencies

The clinical signs of CanL often observed by veterinarians were loss of weight, adenopathy, exfoliative dermatitis and renal disease (Fig. 2). Some veterinarians also described sporadic clinical signs: diarrhoea (*n *= 25), neurological signs (*n *= 5) and lameness (*n *= 18).

Only eight veterinarians described clinical signs in cats testing seropositive for *L. infantum*. The most common of these were: nodular cutaneous lesions, ulcerative lesions, and chronic nasal discharge. In two seropositive cats, a concomitant retrovirus infection was identified.

### Diagnostic methods

**Fig. 3:**
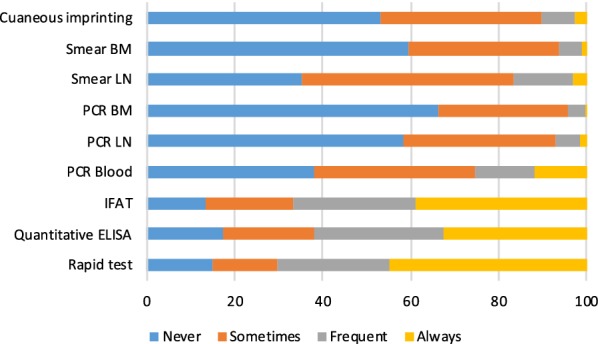
Frequency of use of different diagnostic tools and types of sample

The items of the questionnaire were designed to differentiate between (i) an etiological and immunological diagnosis (serology); and (ii) complementary diagnostic tests (laboratory findings) (Fig. 3).

For an etiological diagnosis, 35.4–59.5% of veterinarians never used cytological tests [lymph node (35.4%), bone marrow (59.5%), cutaneous imprinting (53%)]. Further, 58.4–66.4% also reported they did not carry out PCR techniques on bone marrow or lymph node aspirates, while a blood sample was used by *c.*25% of veterinarians sometimes or even systematically for a PCR diagnosis. Also, some veterinarians used other biological samples such as hair, ear swabs for a PCR diagnosis.

Among the serological techniques used, quantitative methods such as IFAT and ELISA were used always or frequently by 66.7% and 61.8%, respectively. However, responses indicated that qualitative methods such as a rapid test were used as the first diagnostic tool by 70.5%.

**Fig. 4:**
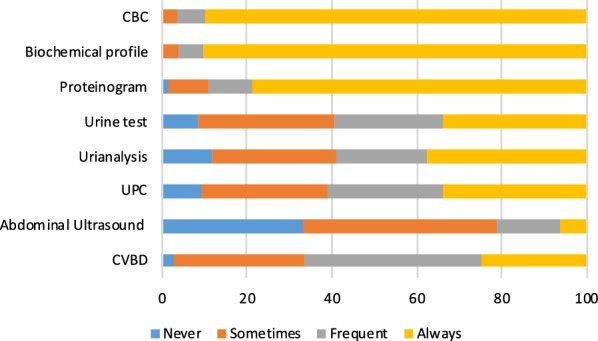
Frequency of use of complementary diagnostic tools

CBC and biochemical profiles were used frequently or systematically by *c.*94% of veterinarians, as well as serum electrophoretogram (89%). This was followed by urinalysis (58.9%) and UPC (61.2%) used frequently or always, although 9.4% and 11.7% had never used UPC and urianalysis, respectively, to assess CanL infection status. Additional proof such as an abdominal ultrasound was frequently used by 21% of veterinarians. Besides, 63.9% of veterinarians diagnosed CanL while conducting tests for other CVBDs (Fig. 4).

### Treatment and follow-up of CanL

**Fig. 5:**
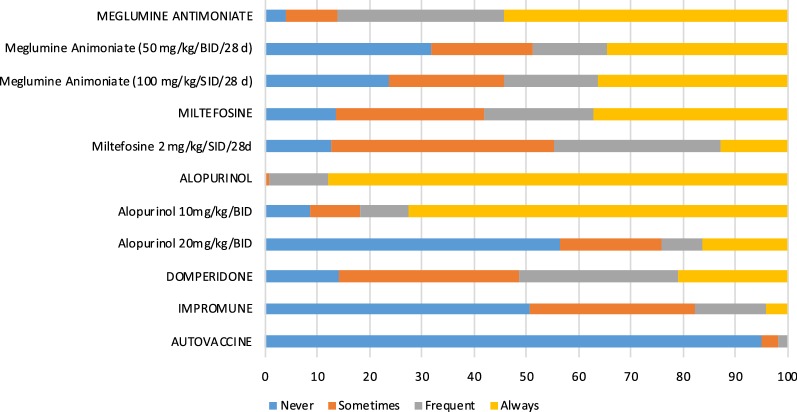
Active principles and frequency of use

More than 90% of veterinarians (54.4% always, 31.9% often and 9.6% sometimes) described they used meglumine antimoniate as treatment for CanL. However, 31.9% and 23.6% stated they had never used the doses recommended by LeishVet of 50 mg/kg/BID/28 d and 100 mg/kg/SID/28 d. Miltefosine was used by around 80% of veterinarians (12.7% always, 31.8% often and 42.7% sometimes), and usually at the dose recommended by the manufacturer. Allopurinol was used systemically practically by 100% of participants, the most common dose being 10 mg/kg BID. Immunomodulators such as domperidone were often or sometimes added by 30.6% and 34.3% of veterinarians, respectively; and systematically used by 20.9%. Regarding the use of Impromune® (Bioibérica, Spain), 50.6% reported they had never used it compared to 4.2% and 13.6% who did so systematically or often, respectively. Finally, 95% of the survey respondents indicated they had never used autovaccine for the treatment of CanL (Fig. 5).

Responses to questions about the frequency of follow-up of dogs with CanL were: visits every 3 or 6 months respectively in 26.8% and 32.3%, and yearly in 5.2%. Responses by 35.7% of veterinarians were that they only scheduled visits if there was clinical recurrence.

### Survival and euthanasia

**Fig. 6:**
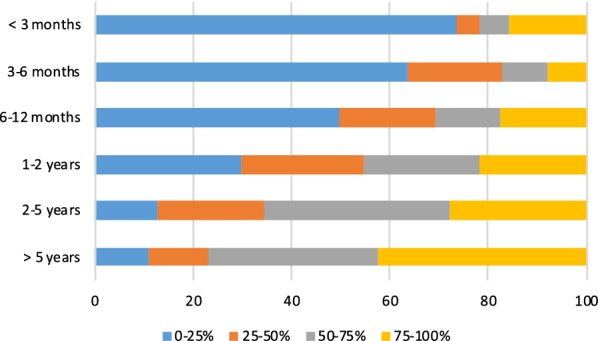
Survival rate of dogs with CanL treated with the different agents

Figure 6 shows the survival rates of dogs with CanL. Few dogs (0–25%) showed a survival of less than 3 months, 6 months or 1 year. While 45.4%, 65.8% and 76.8% of the veterinarians surveyed considered that more than 50% of dogs have a survival rate of more than 1, 2 and 5 years, respectively.

The reasons for euthanasia, both those recommended by the veterinarian or at the request of the owner are indicated in Table 1. Among these, owners usually requested euthanasia because they lived with children, elderly or immunocompromised individuals, and veterinarians often recommended euthanasia when there was chronic renal disease or treatment intolerance.

**Table 1:** Reasons for euthanasia of dogs with CanL

Reason	Owner request (%)	Prescribed by veterinarian (%)
Children at home	90.2	9.8
Elderly at home	87.2	12.8
Immunosuppressed persons at home	66.7	33.3
Living with other dogs	88.6	11.4
Living in shelters or refugees with other dogs	79.3	20.7
Economic	96.0	4.0
Treatment intolerance	28.9	71.1
Chronic renal disease	20.8	78.8
Other concomitant diseases	17.0	83.0

### Preventive measures

**Fig. 7:**
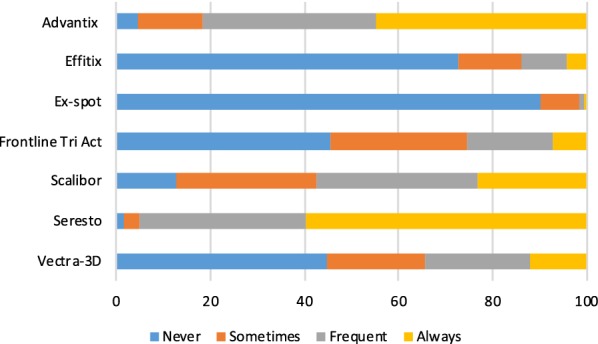
Repellents/insecticides used against sand flies and frequency of use

The surveyed practitioners recommended the following preventive measures: (i) annual serological analysis (80.4%); (ii) use of repellents against the vector (96.7%); (iii) avoiding going outdoors during hours of greater *Phlebotomus* fly activity (66%); (iv) use of mosquito nets (32%); (v) use of domperidone (47.1%); and (vi) vaccination against *L. infantum* (87.6%). Repellents most frequently recommended were: Seresto® (Bayer AnimalHealth, Germany), Advantix® (Bayer AnimalHealth, Germany) and Scalibor® (MSD, France) (Fig. 7).

When asked about vaccines, 87.6% recommended this practice. However, when we asked about the type of vaccine, 22.1% and 69.4% said they frequently or systematically used CanLe

**Table 2:** Responses of veterinarians surveyed regarding vaccination against CanL

Question	Response
Do you recommend vaccination against CanL?	87.6
Yes	87.6
No	12.4
Do you recommend preventive measures in vaccinated dogs?	
Always	99.6
Sometimes	0.4
Do you use CaniLeish®?	
Never	38.7
Rarely	8.9
Sometimes	31.2
Often	18.3
Always	3.8
Do you use LetiFend®?	
Never	8.1
Rarely	0.8
Sometimes	2.5
Often	30.1
Always	39.4
Do you perform a serological test before vaccination?	
Never	3.1
IFAT/ELISA	11.7
Qualitative test	85.2
Do you perform a serological test before re-vaccination?	
Never	33.7
IFAT/ELISA	7.6
Qualitative test	58.7
Do you recommend repellents in vaccinated dogs	
Never	0
Sometimes	0.4
Always	99.6
Have you ever observed any of the following at the inoculation point after vaccination?	
Pain	
Never	37.5
Sometimes	43.0
Often	19.5
Always	0
Erythema	
Never	45.5
Sometimes	43.0
Often	10.6
Always	0.8
Apathy	
Never	57.6
Sometimes	34.3
Often	8.0
Always	0
Digestive disorders	
Never	57.5
Sometimes	33.4
Often	9.0
Always	0
Hypersensitivity	
Never	64.4
Sometimes	30.5
Often	5.1
Always	0

*Note*: All data are expressed as percentages

ish® (Virbac, France) and LetiFend® (Leti, Spain), respectively (Table 2).

When we asked about the detection of antibodies against *L. infantum* before vaccination, 96.9% of the veterinarians said they obtained serology proof before primary vaccination (85.2% undertook rapid tests while 11.7% conducted a quantitative test (IFAT or ELISA), whereas this figure was 66.3% before re-vaccination (58.7% admitted they only used rapid tests, and 7.6% used quantitative tests).

Veterinarians considered that vaccination was safe, as associated clinical signs were not usually observed. The most common clinical signs reported were pain and erythema at the point of inoculation.

### Public health considerations

One of the last questions was whether the veterinarians explained to owners the zoonotic impacts of CanL. Replies were 73.4% always, 12.7% often, 8.7% sometimes and 5.2% rarely.

### What about LeishVet?

Veterinarians surveyed mentioned they knew about the LeishVet research group (64.9%) compared to 35.1% who have never heard about LeishVet.

Of those who were aware of LeishVet, 53.6% had heard of LeishVet in a conference about CanL, 17.8% *via* a veterinary laboratory, 8.9% *via* a veterinary journal, 7.1% *via* an internet search, 6.5% *via* their University and 5.9% *via* a colleague.

When comparing which veterinarians followed LeishVet guideline recommendations on diagnostic tools, treatment and monitoring of sick dogs, preventative measures, and vaccination, significant differences were only detected in the use of abdominal ultrasound as a recommendation (*χ*^2^= 13.643, *df *= 4, *P *= 0.009).

## Discussion

In this survey, we collected information about the clinical management of CanL from 295 practitioners working in 43 Spanish provinces. Despite the large number of surveys sent (*n *= 1428), we consider that the number of veterinarians who answered was low. However, these results are more representative than those of our initial study in 2005 in which data were obtained from only 106 veterinarians across five Spanish provinces [25]. Moreover, in the 2005 study, the veterinarians surveyed worked only in areas showing a high endemicity of CanL, while this time low endemicity areas were also surveyed as in the studies of Bourdeau et al. [26] and Le Rutte et al. [27].

Before asking about CanL, a question was included about other CVBD, and many of the veterinarians indicated they mainly detected CanL and ehrlichiosis. Nevertheless, other CVBD (e.g. dirofilariosis and piroplasmosis) were also reported. According to previous epidemiological studies, the presence of some vector-borne diseases is associated with geographical distribution [28–33]. Indeed, in this survey, dirofilariosis (South of Spain, East of Spain and Canary Islands) and piroplasmosis (North of Spain) were associated with this variable as suggested by others [29, 33–35]. According to these new results, CanL seems to be the most widely CVBD distributed in Spain. However, veterinarians working in areas showing a high or medium endemicity considered that the incidence of CanL remains stable, while those working in low CanL endemic areas indicated that cases of CanL have increased. This perception is similar to that described in other European countries that were until recently free of CanL, such as the UK or Germany [36–39]. The reason for this is unknown, but could be due to (i) the increased transfer of infected dogs from endemic areas, such as rehomed or hunting dogs; (ii) more dogs travelling overseas to *Leishmania*-endemic areas (e.g. taken there by their owners on holiday); (iii) other non-vector routes of transmission such as vertical, venereal, blood transfusion or dog-to-dog [40–43] which may be contributing to the spread of autochthonous cases. The reason could also be a combination of these factors.

The clinical manifestations of CanL vary from subclinical to severe disease. In the present study, weight loss, lymphadenomegaly and exfoliative dermatitis were frequently observed in agreement with the findings of other studies and surveys [25, 26, 44–47]. While renal disease, vasculitis, ocular and join disease have been observed less frequently, these are indicators of a worse prognosis because they are clinical signs related to immunocomplex deposition [48–52]. Additionally, other clinical signs have been sporadically observed such as neurological signs [53–57] or digestive disorders [58–60]. Although these signs could due to CanL, other diseases must be considered in the differential diagnosis.

Regarding the diagnosis questions, the present survey differentiated between an etiological diagnosis (microscopy observation and/or PCR), serology and complementary tests. It should be noted that more than 35% of the practitioners surveyed mentioned they had never performed an etiological diagnosis, and if this was done, the most common method used was PCR on blood samples or cytology on lymph node aspirates. Cytology (5.3%) or PCR (3.9%) on bone marrow aspirates were infrequently used for an etiological diagnosis.

Similar results have been obtained in questionnaire surveys by other authors [26, 46]. However, according to the LeishVet group, blood is one of the least sensitive biological samples, and the use of bone marrow or lymph node aspirates is strongly recommended [2, 61]. Bourdeau et al. [26] suggested veterinarians could reject the more invasive techniques due to the extra costs and the fact they are time-consuming as in some cases it will be necessary to sedate the animal. Other authors suggest a lack of referral laboratories close to veterinary practices surveyed as the reason for the reduced use of molecular techniques [62]. However, we found no difference in the approach used according to the location of the veterinary practice.

Quantitative serological diagnosis is essential in the diagnosis and monitoring of CanL, and although many veterinarians used the IFAT technique (66.7%) and quantitative ELISA (51.8%) frequently or systematically, there were also many clinics that used rapid tests (70.5%) as the first diagnostic approach, in agreement with the results of similar surveys [25, 26, 63]. While a rapid test may help confirm clinically suspected cases as they show high specificity in sick dogs, the indirect fluorescent antibody test and the enzyme-linked immunosorbent assay are the most suitable serological tools according the LeishVet group and OIE [2, 64], as confirmed by several authors [65–67]. In other surveys conducted in Spain and other CanL endemic countries (e.g. Italy), IFAT was considered the gold standard by veterinary practices surveyed [45–47].

According to the responses, almost all veterinarians used CBC and biochemical profiles to monitor infected dogs, and *c.*80% also used electrophoretograms. These rates are significantly higher than those of our previous survey [25], and indicate the improved clinical management of CanL as suggested by the LeishVet group [2, 21]. Regarding the use of urinalysis, ranges of 25.5–33.9% to 21.5–37.4% used urine strip and urinalysis, respectively. Also, 27.2–34.0% frequently or systematically, respectively, determined the UPC. This is important, as according to LeishVet and IRIS guidelines, UPC is a highly suitable biomarker for diagnosis of CanL because immunocomplex deposits in the renal glomerulus may induce immune-mediated glomerulonephritis. In sick dogs, this serious kidney lesion causes proteinuria with or without azotemia and chronic renal disease or even nephrotic syndrome of worse prognosis [68, 69]. Another important complementary diagnosis is abdominal ultrasound which was performed frequently during CanL follow-up by 6.4–14.6% of the veterinarians surveyed. These diagnostic methods (UPC and ultrasound) were not included in other surveys but the present responses suggest that veterinarians have access to this new information on the clinical management of CanL *via* the LeishVet guidelines, IRIS guidelines and specialized conferences [2, 21, 69, 70].

The LeishVet guidelines suggest that CanL therapy should be based on clinical staging (serological status, clinical signs and laboratory findings), and recommended regimens are the combination of allopurinol and meglumine antimoniate or miltefosine in dogs with disease stage II or III [2, 21]. The results of the present survey suggest veterinarians frequently use the combination of meglumine antimoniate and allopurinol, followed by miltefosine, in accordance with a previous questionnaire survey [47]. The long-term use of allopurinol, in combination with n-methylglucamine antimoniate or miltefosine, has proved to be effective for maintaining clinical and parasitological improvement and delaying relapses in treated dogs. Miltefosine is better tolerated with regard to liver and kidney function. There is scarce information regarding possible toxicity of antimonials, and some authors have suggested possible kidney and liver secondary effects [71–73]. It is also true, however, that disease relapse occurs much earlier in dogs treated with miltefosine [73, 74]. Therefore, although available data are controversial, miltefosine could be recommended for treatment of canine leishmaniosis patients with renal or liver disease, and/or when subcutaneous or parenteral therapy may not be administered or is not indicated [73–76].

When veterinarians were asked about regimens of n-methylglucamine antimoniate, they indicated the 100 mg/kg/SID/28 d dose was more used than 50 mg/kg/BID/28 d, although differences were not significant. This could be related to the fact that some owners prefer to inject their dogs once a day rather than twice.

Most veterinarians stated they used allopurinol in combination with leishmanicide drugs, as allopurinol has been shown to prevent recurrence [77]. Likewise, it has been shown that dogs treated with allopurinol are not able to transmit the parasite to sand flies [78]. However, some dogs develop xanthinuria due to the inhibition of the enzyme xanthine oxidase, so it is strongly recommended to include frequent urinalysis and abdominal ultrasound for monitoring dogs under long term therapy with allopurinol [79], and assess renal mineralisation and urolithiasis [80]. The veterinarians surveyed responded they used urinalysis (58.9–61.2%) and ultrasound (21%) as preventative of adverse urinary effects.

Avoiding sand fly bites is one of the best ways to stop the spread of *L. infantum* infection [22, 81]. Results obtained in this survey indicate that veterinarians recommend the use of repellents against the vector (96.7%), mainly collars and spot-ons as in other studies [45, 62, 82]. Other measures to avoid sand fly bites were not often recommended such as avoiding dogs going outside during hours of sand fly activity (from dusk to dawn) and using mosquito nets. These preventive measures have increased in comparison to the 2005 survey [25]. Sixty-six percent of veterinarians recommended keeping dogs indoors during the risk period and 32% recommended the use of mosquito nets.

Lastly, another preventative measure recommended was vaccination. In the 2005 survey, 100% of veterinarians asked about vaccination answered that if there were a vaccine available, they would use it. In this survey, 87.6% replied they recommended vaccination as a preventative measure. Further, it is remarkable that the most common vaccine used by veterinarians surveyed is LetiFend® and not Canileish®. This lower use than initially expected could be because its percentage effectiveness is *c.*70% [83]. In addition, Canileish® needs to be given for primovaccination as three consecutive doses three weeks apart to develop an adequate immune response while LetiFend® is a single dose even for primary vaccination. This vaccine is also a DIVA (Differentiating Infected from Vaccinated Animals) vaccine, allowing to discriminate vaccinated from infected dogs. For all these reasons, LetiFend® is at the first-line vaccine option over Canileish®, as reflected in the results of other surveys [84–86].

When asked about adverse reactions associated with vaccination, while most veterinarians consider them safe enough, adverse reactions to vaccination are mentioned in the vaccineʼs Summary of Product Characteristics (SPC). In experimental trials by Oliva et al. [24] and Lemesre et al. [87] no signs of a local or systemic reaction were observed one week after vaccination [24, 87]. In contrast, Lladró et al. [44] found, through a questionnaire completed by 45 practitioners in Girona (NE Spain), that 82% of veterinarians mentioned adverse reactions (local reaction, gastrointestinal signs, fever, apathy, vasovagal syncope and anaphylactic shock) potentially associated with vaccination [44]. Further, to date, no dogs receiving LetiFend® have shown any associated local or systemic adverse events [86, 88]. However, in our survey the appearance of clinical signs could be correlated with the vaccine used (CaniLeish® or LetiFend®).

Vaccine manufacturers recommend performing a serological test before vaccination. In this survey almost all veterinarians mentioned they undertook a pre-primovaccination test. However, a high percentage of veterinarians stated they never performed any test before revaccination so it cannot be known if a dog has been infected during the inter-vaccination period. This test is strictly necessary since vaccines do not protect 100% [83]. On the other hand, the results of the present survey indicate that more veterinarians carry out rapid tests before vaccination. Although rapid tests have a good sensitivity and specificity, quantitative techniques are considered more sensitive. Thus, a quantitative test is always recommended before vaccination to ensure the vaccination of healthy animals, as sometimes dogs with low antibody titres are not detected using qualitative techniques [83, 89] due to their lower sensitivity.

Current treatments and follow-up have allowed for a greater survival of dogs with CanL compared to the results of the 2005 survey [25], over 50% of dogs showing a survival longer than 5 years. However, in some cases, a veterinarian will recommend euthanasia for humanitarian reasons, especially when there is renal disease or other associated diseases. According to the present survey, an owner will usually request euthanasia when there are small children, or immunocompromised or elderly persons at home, and when a dog lives in a shelter. The educational role of veterinarians is essential to explain the life-cycle of the parasite and how other animals or people could acquire the infection, highlighting the importance of control and prevention measures [21, 22]. Veterinarians also need to make owners aware that culling CanL positive dogs is not an adequate disease control measure, as confirmed in studies conducted in Brazil where culling seropositive dogs failed to reduce the incidence of canine or human leishmaniosis [90–92].

Another aspect to consider is that the dog is not the only reservoir capable of transmitting *L. infantum*. Several studies have shown that *Leishmania-*competent vectors feed on cats naturally infected with *L. infantum* and through xenodiagnosis have confirmed that infected cats can transmit the infection to *P. perniciosus* and *L. longipalpis* [93–95]. However, in endemic areas of CanL, subclinical feline infections are common, although clinical cases are so far exceptional [18, 96, 97]. Indeed, in the present questionnaire, 8% cases of feline leishmaniosis were reported although these data are insufficient to reveal the role of cats as a possible reservoir as the veterinarians did not specify the diagnosis technique or clinical signs.

The LeishVet group is a scientific association focusing on offering a consensus statement on the clinical management of CanL and standardizing criteria for its diagnosis and treatment. The results of the present survey indicate that around 65% of the surveyed practitioners knew about LeishVet. This contradicts the data collected in the questionnaire of Le Rutte et al. [27], in which 73.5% of the Spanish veterinarians surveyed were not aware of any guidelines of canine leishmaniosis. Further, we may assume that currently veterinarians have adequate information about CanL as almost all the replies obtained in this survey were in line with LeishVet guidelines.

## Conclusions

Despite significant changes in the clinical management of CanL, its incidence has not decreased in recent years, due to factors such as improved clinical knowledge of the disease on the part of veterinarians, better available diagnostic techniques and their adequate interpretation, as well as a greater awareness of owners of control measures available (repellents and vaccines). We should thus continue to well inform practitioners so that they can improve health education among dog owners.

## Supplementary information

**Additional file 1: Text S1.** Questionnaire that was distributed to veterinarians.

## Data Availability

Data supporting the conclusions of this article are included within the article.

## Abbreviations

BID: twice a day
CanL: canine leishmaniosis
CBC: complete blood count
CVBD: canine vector borne diseases
d: day
DIVA: differentiating infected from vaccinated animals
ELISA: enzyme-linked immunosorbent assay
FelL: feline leishmaniosis
IFAT: indirect immunofluorescence antibody test
IRIS: International Renal Interest Society
PCR: polymerase chain reaction
SID: once a day
UPC: urine protein/creatinine ratio
VBD: vector borne diseases

## References

[1] Alvar J, Cañavate C, Molina R, Moreno J, Nieto J (2004). Canine leishmaniasis. Adv Parasitol.

[2] Solano-Gallego L, Miró G, Koutinas A, Cardoso L, Pennisi MG, Ferrer L (2011). LeishVet guidelines for the practical management of canine leishmaniosis. Parasites Vectors.

[3] Gramiccia M, Gradoni L (2005). The current status of zoonotic leishmaniases and approaches to disease control. Int J Parasitol.

[4] Millán J, Ferroglio E, Solano-Gallego L (2014). Role of wildlife in the epidemiology of *Leishmania infantum* infection in Europe. Parasitol Res.

[5] Baneth G, Thamsborg SM, Otranto D, Guillot J, Blaga R, Deplazes P (2016). Major parasitic zoonoses associated with dogs and cats in Europe. J Comp Pathol.

[6] Navarro JA, Sánchez J, Peñafiel-Verdú C, Buendía AJ, Altimira J, Vilafranca M (2010). Histopathological lesions in 15 cats with leishmaniosis. J Comp Pathol.

[7] Martín-Sánchez J, Acedo C, Muñoz-Pérez M, Pesson B, Marchal O, Morillas-Márquez F (2007). Infection by *Leishmania infantum* in cats: epidemiological study in Spain. Vet Parasitol.

[8] Arce A, Estirado A, Ordobas M, Sevilla S, García N, Moratilla L (2013). Re-emergence of leishmaniasis in Spain: community outbreak in Madrid, Spain, 2009 to 2012. Euro Surveill.

[9] Molina R, Jiménez MI, Cruz I, Iriso A, Martín-Martín I, Sevillano O (2012). The hare (*Lepus granatensis*) as potential sylvatic reservoir of *Leishmania infantum* in Spain. Vet Parasitol.

[10] García N, Moreno I, Alvarez J, de la Cruz ML, Navarro A, Pérez-Sancho M (2014). Evidence of *Leishmania infantum* infection in rabbits (*Oryctolagus cuniculus*) in a natural area in Madrid, Spain. Biomed Res Int.

[11] Luppi MM, Malta MC, Silva TM, Silva FL, Motta RO, Miranda I (2008). Visceral leishmaniasis in captive wild canids in Brazil. Vet Parasitol.

[12] Quinnell RJ, Courtenay O (2009). Transmission, reservoir hosts and control of zoonotic visceral leishmaniasis. Parasitology.

[13] Malta MC, Tinoco HP, Xavier MN, Vieira AL, Costa EA, Santos RL (2010). Naturally acquired visceral leishmaniasis in non-human primates in Brazil. Vet Parasitol.

[14] Aguirre AA (2009). Essential veterinary education in zoological and wildlife medicine: a global perspective. Rev Sci Tech.

[15] Scotch M, Odofin L, Rabinowitz P (2009). Linkages between animal and human health sentinel data. BMC Vet Res.

[16] Miró G, López-Vélez R (2018). Clinical management of canine leishmaniosis *versus* human leishmaniasis due to *Leishmania infantum*: putting “One Health” principles into practice. Vet Parasitol.

[17] Alonso F, Giménez Font P, Manchón M, Ruiz de Ybáñez R, Segovia M, Berriatua E (2010). Geographical variation and factors associated to seroprevalence of canine leishmaniosis in an endemic Mediterranean area. Zoonoses Public Health..

[18] Pennisi MG, Cardoso L, Baneth G, Bourdeau P, Koutinas A, Miró G (2015). LeishVet update and recommendations on feline leishmaniosis. Parasites Vectors.

[19] Daza Gonzalez MA, Fragio Arnold C, Fermin Rodriguez M, Checa R, Montoya A, Portero Fuentes M (2019). Effect of two treatments on changes in serum acute phase protein concentrations in dogs with clinical leishmaniosis. Vet J.

[20] García-Martínez JD, Tvarijonaviciute A, Cerón JJ, Caldin M, Martínez-Subiela S (2012). Urinary clusterin as a renal marker in dogs. J Vet Diagn Invest.

[21] Miró G, Petersen C, Cardoso L, Bourdeau P, Baneth G, Solano-Gallego L (2017). Novel areas for prevention and control of canine leishmaniosis. Trends Parasitol.

[22] Gálvez R, Montoya A, Fontal F, Martínez De Murguía L, Miró G (2018). Controlling phlebotomine sand flies to prevent canine *Leishmania infantum* infection: a case of knowing your enemy. Res Vet Sci.

[23] Gradoni L (2015). Canine *Leishmania* vaccines: still a long way to go. Vet Parasitol.

[24] Oliva G, Nieto J, Foglia Manzillo V, Cappiello S, Fiorentino E, Di Muccio T (2014). A randomised, double-blind, controlled efficacy trial of the LiESP/QA-21 vaccine in naïve dogs exposed to two *Leishmania infantum* transmission seasons. PLoS Negl Trop Dis.

[25] Miró G, Molina R (2006). Leishmaniosis canina: manejo clínico y situación actual en España.

[26] Bourdeau P, Saridomichelakis MN, Oliveira A, Oliva G, Kotnik T, Gálvez R (2014). Management of canine leishmaniosis in endemic SW European regions: a questionnaire-based multinational survey. Parasites Vectors.

[27] Le Rutte EA, van Straten R, Overgaauw PAM (2018). Awareness and control of canine leishmaniosis: a survey among Spanish and French veterinarians. Vet Parasitol.

[28] Beugnet F, Marié JL (2009). Emerging arthropod-borne diseases of companion animals in Europe. Vet Parasitol.

[29] Solano-Gallego L, Sainz Á, Roura X, Estrada-Peña A, Miró G (2016). A review of canine babesiosis: the European perspective. Parasites Vectors.

[30] Ready PD (2010). Leishmaniasis emergence in Europe. Euro Surveill.

[31] Ready PD (2008). Leishmaniasis emergence and climate change. Rev Sci Tech.

[32] Genchi C, Mortarino M, Rinaldi L, Cringoli G, Traldi G, Genchi M (2011). Changing climate and changing vector-borne disease distribution: the example of *Dirofilaria* in Europe. Vet Parasitol.

[33] Simón L, Afonin A, López-Díez LI, González-Miguel J, Morchón R, Carretón E (2014). Geo-environmental model for the prediction of potential transmission risk of *Dirofilaria* in an area with dry climate and extensive irrigated crops. The case of Spain. Vet Parasitol.

[34] Miro G, Montoya A, Roura X, Galvez R, Sainz A (2013). Seropositivity rates for agents of canine vector-borne diseases in Spain: a multicentre study. Parasites Vectors.

[35] Checa R, Fidalgo LE, Montoya A, Lopez AM, Barrera JP, Galvez R (2019). The role of healthy dog carriers of *Babesia microti*-like piroplasms. Parasites Vectors.

[36] Fooks AR, Johnson N (2015). Jet set pets: examining the zoonosis risk in animal import and travel across the European Union. Vet Med.

[37] Maia C, Cardoso L (2015). Spread of *Leishmania infantum* in Europe with dog travelling. Vet Parasitol.

[38] Menn B, Lorentz S, Naucke TJ (2010). Imported and travelling dogs as carriers of canine vector-borne pathogens in Germany. Parasites Vectors.

[39] Schäfer I, Volkmann M, Beelitz P, Merle R, Müller E, Kohn B (2019). Retrospective evaluation of vector-borne infections in dogs imported from the Mediterranean region and southeastern Europe (2007–2015). Parasites Vectors.

[40] Vida B, Toepp A, Schaut RG, Esch KJ, Juelsgaard R, Shimak RM (2016). Immunologic progression of canine leishmaniosis following vertical transmission in United States dogs. Vet Immunol Immunopathol.

[41] Duprey ZH, Steurer FJ, Rooney JA, Kirchhoff LV, Jackson JE, Rowton ED (2006). Canine visceral leishmaniasis, United States and Canada, 2000–2003. Emerg Infect Dis.

[42] Silva FL, Oliveira RG, Silva TM, Xavier MN, Nascimento EF, Santos RL (2009). Venereal transmission of canine visceral leishmaniasis. Vet Parasitol.

[43] Naucke TJ, Amelung S, Lorentz S (2016). First report of transmission of canine leishmaniosis through bite wounds from a naturally infected dog in Germany. Parasites Vectors.

[44] Lladró S, Picado A, Ballart C, Portús M, Gállego M (2017). Management, prevention and treatment of canine leishmaniosis in north-eastern Spain: an online questionnaire-based survey in the province of Girona with special emphasis on new preventive methods (CaniLeish vaccine and domperidone). Vet Rec.

[45] Gálvez R, Miró G, Descalzo MA, Molina R (2011). Questionnaire-based survey on the clinical management of canine leishmaniosis in the Madrid region (central Spain). Prev Vet Med.

[46] de Ybáñez RR, del Río L, Martínez-Carrasco C, Segovia M, Cox J, Davies C (2009). Questionnaire survey on canine leishmaniosis in southeastern Spain. Vet Parasitol.

[47] Morosetti G, Bongiorno G, Beran B, Scalone A, Moser J, Gramiccia M (2009). Risk assessment for canine leishmaniasis spreading in the north of Italy. Geospat Health.

[48] Goto H, Prianti M (2009). Immunoactivation and immunopathogeny during active visceral leishmaniasis. Rev Inst Med Trop Sao Paulo.

[49] Koutinas AF, Koutinas CK (2014). Pathologic mechanisms underlying the clinical findings in canine leishmaniasis due to *Leishmania infantum/chagasi*. Vet Pathol.

[50] Parody N, Cacheiro-Llaguno C, Osuna C, Renshaw-Calderón A, Alonso C, Carnés J (2019). Circulating immune complexes levels correlate with the progression of canine leishmaniosis in naturally infected dogs. Vet Parasitol.

[51] Pumarola M, Brevik L, Badiola J, Vargas A, Domingo M, Ferrer L (1991). Canine leishmaniasis associated with systemic vasculitis in two dogs. J Comp Pathol.

[52] García-Alonso M, Blanco A, Reina D, Serrano FJ, Alonso C, Nieto CG (1996). Immunopathology of the uveitis in canine leishmaniasis. Parasite Immunol.

[53] Zobba R, Evangelisti MA, Manunta ML, Alberti A (2017). A case of canine neurological leishmaniasis. Vet Ital.

[54] Viñuelas J, García-Alonso M, Ferrando L, Navarrete I, Molano I, Mirón C (2001). Meningeal leishmaniosis induced by *Leishmania infantum* in naturally infected dogs. Vet Parasitol.

[55] Márquez M, Pedregosa JR, López J, Marco-Salazar P, Fondevila D, Pumarola M (2013). *Leishmania* amastigotes in the central nervous system of a naturally infected dog. J Vet Diagn Invest.

[56] José-López R, la Fuente CD, Añor S (2012). Presumed brain infarctions in two dogs with systemic leishmaniasis. J Small Anim Pract.

[57] José-López R, de la Fuente C, Pumarola M, Añor S (2014). Intramedullary spinal cord mass presumptively associated with leishmaniasis in a dog. J Am Vet Med Assoc.

[58] Ayala I, Bernal LJ, Garcia-Martinez JD, Gomez MA, Navarro JA, Bernabe A (2017). An atypical case of leishmaniasis associated with chronic duodenitis in a dog. J Am Anim Hosp Assoc.

[59] Ruiz G, Laloy E, Benchekroun G (2015). Chronic gastritis and enterocolitis associated with *Leishmania* infection in an 18-month-old, intact female dog. Vet Q.

[60] Ferrer L, Juanola B, Ramos JA, Ramis A (1991). Chronic colitis due to *Leishmania* infection in two dogs. Vet Pathol.

[61] Hernandez L, Montoya A, Checa R, Dado D, Galvez R, Otranto D (2015). Course of experimental infection of canine leishmaniosis: follow-up and utility of noninvasive diagnostic techniques. Vet Parasitol.

[62] Alcover MM, Ballart C, Serra T, Castells X, Scalone A, Castillejo S (2013). Temporal trends in canine leishmaniosis in the Balearic Islands (Spain): a veterinary questionnaire. Prospective canine leishmaniosis survey and entomological studies conducted on the Island of Minorca, 20 years after first data were obtained. Acta Trop.

[63] Fernandez M, Tabar MD, Arcas A, Mateu C, Homedes J, Roura X (2018). Comparison of efficacy and safety of preventive measures used against canine leishmaniasis in southern European countries: longitudinal retrospective study in 1647 client-owned dogs (2012–2016). Vet Parasitol.

[64] OIE. Manual Terrestre de la OIE. Capitulo 3.1.11. Leishmaniasis. 2018.

[65] Solano-Gallego L, Villanueva-Saz S, Carbonell M, Trotta M, Furlanello T, Natale A (2014). Serological diagnosis of canine leishmaniosis: comparison of three commercial ELISA tests (Leiscan, ID Screen and Leishmania 96), a rapid test (Speed Leish K) and an in-house IFAT. Parasites Vectors.

[66] Proverbio D, Spada E, Baggiani L, Bagnagatti De Giorgi G, Perego R (2013). Comparison of a clinic-based ELISA test kit with the immunofluorescence antibody test for assaying *Leishmania infantum* antibodies in dogs. Biomed Res Int.

[67] Mettler M, Grimm F, Capelli G, Camp H, Deplazes P (2005). Evaluation of enzyme-linked immunosorbent assays, an immunofluorescent-antibody test, and two rapid tests (immunochromatographic-dipstick and gel tests) for serological diagnosis of symptomatic and asymptomatic *Leishmania* infections in dogs. J Clin Microbiol.

[68] Costa FA, Goto H, Saldanha LC, Silva SM, Sinhorini IL, Silva TC (2003). Histopathologic patterns of nephropathy in naturally acquired canine visceral leishmaniasis. Vet Pathol.

[69] Littman MP, Daminet S, Grauer GF, Lees GE, van Dongen AM, Subgroup ICGSGD (2013). Consensus recommendations for the diagnostic investigation of dogs with suspected glomerular disease. J Vet Intern Med.

[70] Brown S, Elliott J, Francey T, Polzin D, Vaden S, Subgroup ICGSGST (2013). Consensus recommendations for standard therapy of glomerular disease in dogs. J Vet Intern Med.

[71] Daza González MA, Miró G, Fermín Rodríguez M, Rupérez Noguer C, Fragío Arnold C (2019). Short term impacts of meglumine antimoniate treatment on kidney function in dogs with clinical leishmaniosis. Res Vet Sci.

[72] Ikeda-Garcia FA, Lopes RS, Ciarlini PC, Marques FJ, Lima VMF, Perri SHV (2007). Evaluation of renal and hepatic functions in dogs naturally infected by visceral leishmaniasis submitted to treatment with meglumine antimoniate. Res Vet Sci.

[73] Barrientos MM (2007). Estudios sobre la eficacia comparada y la tolerancia de la miltefosina y el antimoniato de n-metilglucamina, y la monitorización post-tratamiento con alopurinol en la infección natural por “*Leishmania infantum*” en el perro.

[74] Manna L, Corso R, Galiero G, Cerrone A, Muzj P, Gravino AE (2015). Long-term follow-up of dogs with leishmaniosis treated with meglumine antimoniate plus allopurinol *versus* miltefosine plus allopurinol. Parasites Vectors.

[75] Manna L, Vitale F, Reale S, Picillo E, Neglia G, Vescio F (2009). Study of efficacy of miltefosine and allopurinol in dogs with leishmaniosis. Vet J.

[76] Noli C, Saridomichelakis MN (2014). An update on the diagnosis and treatment of canine leishmaniosis caused by *Leishmania infantum* (syn. *L. chagasi*). Vet J.

[77] Noli C, Auxilia ST (2005). Treatment of canine Old World visceral leishmaniasis: a systematic review. Vet Dermatol.

[78] Miró G, Gálvez R, Fraile C, Descalzo MA, Molina R (2011). Infectivity to *Phlebotomus perniciosus* of dogs naturally parasitized with *Leishmania infantum* after different treatments. Parasites Vectors.

[79] Miró G. Tratamiento y pronóstico. In: Leishmaniosis: una revisión actualizada. Zaragoza (Spain): Servet. Grupo Asís; 2013. p. 151–64.

[80] Torres M, Pastor J, Roura X, Tabar MD, Espada Y, Font A (2016). Adverse urinary effects of allopurinol in dogs with leishmaniasis. J Small Anim Pract.

[81] Miró G, Cardoso L, Pennisi MG, Oliva G, Baneth G (2008). Canine leishmaniosis-new concepts and insights on an expanding zoonosis: part two. Trends Parasitol.

[82] Ballart C, Alcover MM, Picado A, Nieto J, Castillejo S, Portús M (2013). First survey on canine leishmaniasis in a non classical area of the disease in Spain (Lleida, Catalonia) based on a veterinary questionnaire and a cross-sectional study. Prev Vet Med.

[83] Solano-Gallego L, Cardoso L, Pennisi MG, Petersen C, Bourdeau P, Oliva G (2017). Diagnostic challenges in the era of canine *Leishmania infantum* vaccines. Trends Parasitol.

[84] Moreno J, Vouldoukis I, Schreiber P, Martin V, McGahie D, Gueguen S (2014). Primary vaccination with the LiESP/QA-21 vaccine (CaniLeish) produces a cell-mediated immune response which is still present 1 year later. Vet Immunol Immunopathol.

[85] Martin V, Vouldoukis I, Moreno J, McGahie D, Gueguen S, Cuisinier AM (2014). The protective immune response produced in dogs after primary vaccination with the LiESP/QA-21 vaccine (CaniLeish®) remains effective against an experimental challenge one year later. Vet Res.

[86] Fernández Cotrina J, Iniesta V, Monroy I, Baz V, Hugnet C, Marañon F (2018). A large-scale field randomized trial demonstrates safety and efficacy of the vaccine LetiFend® against canine leishmaniosis. Vaccine.

[87] Lemesre JL, Holzmuller P, Gonçalves RB, Bourdoiseau G, Hugnet C, Cavaleyra M (2007). Long-lasting protection against canine visceral leishmaniasis using the LiESAp-MDP vaccine in endemic areas of France: double-blind randomised efficacy field trial. Vaccine.

[88] Toepp A, Larson M, Grinnage-Pulley T, Bennett C, Anderson M, Parrish M (2018). Safety analysis of *Leishmania* vaccine used in a randomized canine vaccine/immunotherapy trial. Am J Trop Med Hyg.

[89] Rodríguez-Cortés A, Ojeda A, Todolí F, Alberola J (2013). Performance of commercially available serological diagnostic tests to detect *Leishmania infantum* infection on experimentally infected dogs. Vet Parasitol.

[90] Dantas-Torres F, Miró G, Baneth G, Bourdeau P, Breitschwerdt E, Capelli G (2019). Canine leishmaniasis control in the context of One Health. Emerg Infect Dis.

[91] Dantas-Torres F, Miró G, Bowman DD, Gradoni L, Otranto D (2019). Culling dogs for zoonotic visceral leishmaniasis control: the wind of change. Trends Parasitol.

[92] Sousa-Paula LC, Silva LGD, Sales KGDS, Dantas-Torres F (2019). Failure of the dog culling strategy in controlling human visceral leishmaniasis in Brazil: a screening coverage issue?. PLoS Negl Trop Dis.

[93] Maroli M, Pennisi MG, Di Muccio T, Khoury C, Gradoni L, Gramiccia M (2007). Infection of sandflies by a cat naturally infected with *Leishmania infantum*. Vet Parasitol.

[94] da Silva SM, Rabelo PF, Gontijo NeF, Ribeiro RR, Melo MN, Ribeiro VM (2010). First report of infection of *Lutzomyia longipalpis* by *Leishmania* (*Leishmania*) *infantum* from a naturally infected cat of Brazil. Vet Parasitol.

[95] Afonso MM, Duarte R, Miranda JC, Caranha L, Rangel EF (2012). Studies on the feeding habits of *Lutzomyia* (*Lutzomyia*) *longipalpis* (Lutz & Neiva, 1912) (Diptera: Psychodidae: Phlebotominae) populations from endemic areas of american visceral leishmaniasis in northeastern Brazil. J Trop Med.

[96] Miro G, Ruperez C, Checa R, Galvez R, Hernandez L, Garcia M (2014). Current status of *L. infantum* infection in stray cats in the Madrid region (Spain): implications for the recent outbreak of human leishmaniosis?. Parasites Vectors.

[97] Montoya A, Garcia M, Galvez R, Checa R, Marino V, Sarquis J (2018). Implications of zoonotic and vector-borne parasites to free-roaming cats in central Spain. Vet Parasitol.

