# Supplementary material for: Latest trends in L. infantum infection in dogs in Spain, Part II: current clinical management and control according to a national survey of veterinary practitioners
Source: Parasit Vectors. 2020 Apr 21;13:205. doi: 10.1186/s13071-020-04080-8 (PMC7175573; doi:10.1186/s13071-020-04080-8)
Supplement: Supplementary file 1 — Additional file 1: Text S1. Questionnaire that was distributed to veterinarians. [file 13071_2020_4080_MOESM1_ESM.docx]

**Additional file 1: Text S1**

**1. Indicate the province of your small animal veterinarian practice ____________**

2. **How many veterinarians work at the clinic?**

- 2 veterinarians
- 3-4 veterinarians
- Other (specify) **_________________**

**3. How many animals do you see per day?**

- <5 animals
- 5-10 animals
- >10 animals

4. **Indicate which of the following vector diseases you diagnose most frequently**

* Select all those that apply

- Leishmaniosis
- Dirofilariosis
- Ehrlichiosis
- Piroplasmosis
- Anaplasmosis
- Other (specify) **_________________**

**Regarding canine leishmaniosis**

5. **How many new clinical cases do you diagnose each year?**

- Fewer than 5 cases
- 5-10 cases
- More than 10 cases
- Annual estimate (specify):**_________________**

6. **What would you say is the trend in the incidence of leishmaniosis in your area (in the past 5 years)?**

- It has decreased
- It has remained stable
- It has increased

7. **What is the clinical picture that you observe most frequently?**

|  | Never | Sometimes Often Always | Often | Always |
| --- | --- | --- | --- | --- |
| Weight loss |  |  |  |  |
| Lymphadenomegaly |  |  |  |  |
| Asthenia |  |  |  |  |
| Pale mucous membranes |  |  |  |  |
| Exfoliative dermatitis |  |  |  |  |
| Ulcerative forms |  |  |  |  |
| Vasculitis |  |  |  |  |
| Onycogryphosis |  |  |  |  |
| Uveitis |  |  |  |  |
| Epistaxis |  |  |  |  |
| Renal disease |  |  |  |  |
| Others (specify): |  |  |  |  |

8. **How do you diagnose the disease?**

|  | Never | Sometimes Often Always | Often | Always |
| --- | --- | --- | --- | --- |
| SMEAR AND MICROSCOPY |  |  |  |  |
| Cutaneous imprint |  |  |  |  |
| Bone marrow aspirate |  |  |  |  |
| Lymph node aspirate |  |  |  |  |
| QUANTITATIVE SEROLOGY |  |  |  |  |
| IFAT |  |  |  |  |
| ELISA |  |  |  |  |
| QUALITATIVE SEROLOGY |  |  |  |  |
| Rapid tests |  |  |  |  |
| PCR on: |  |  |  |  |
| Blood |  |  |  |  |
| Lymph node aspirate |  |  |  |  |
| Bone marrow aspirate |  |  |  |  |
| Other biological samples (specify): |  |  |  |  |

9. **Indicate the complementary tests that you usually perform**

|  | Never | Sometimes Often Always | Often | Always |
| --- | --- | --- | --- | --- |
| CBC |  |  |  |  |
| Biochemical profile |  |  |  |  |
| Electrophoretogram |  |  |  |  |
| Urine strip |  |  |  |  |
| Urinalysis |  |  |  |  |
| Urine protein/creatinine ratio (UPC) |  |  |  |  |
| Abdominal ultrasound |  |  |  |  |
| Others (specify): |  |  |  |  |

10. **Indicate the active ingredients you use to treat the disease**

|  | Never | Sometimes Often Always | Often | Always |
| --- | --- | --- | --- | --- |
| Antimonials |  |  |  |  |
| Miltefosine |  |  |  |  |
| Allopurinol |  |  |  |  |
| Domperidone |  |  |  |  |
| Nucleotides (Impromune®)(Bioibérica) |  |  |  |  |
| Others (specify): |  |  |  |  |

11. **Which regimen do you use to treat canine leishmaniosis?**

|  | Never | Sometimes Often Always | Often | Always |
| --- | --- | --- | --- | --- |
| Antimoniales |  |  |  |  |
| 50 mg/kg/12 h/4 w(s.c.) |  |  |  |  |
| 100 mg/kg/24 h/4 w (s.c.) |  |  |  |  |
| Other (specify): |  |  |  |  |
| Miltefosine |  |  |  |  |
| 2 mg/kg/24 h/ 4 w |  |  |  |  |
| Other (specify): |  |  |  |  |
| Allopurinol |  |  |  |  |
| 10 mg/kg/12h/6-12 months |  |  |  |  |
| 20 mg/kg/24 h/6-12 months |  |  |  |  |
| Other (specify): |  |  |  |  |
| Domperidone (0.5 mg/kg/d) |  |  |  |  |
| Specify: |  | | | |
| Nucleotides (Impromune®) (Bioibérica) |  |  |  |  |
| Specify: |  |  |  |  |
| Other (specify): |  |  |  |  |

1. **Regarding disease follow-up, how often do you carry out clinical check-ups?**

- Every 3 months
- Every 6 months
- Once a year
- Other (specify)

1. **According to your experience, what is the survival rate of treated dogs?**

|  | **0-25%** | **25-50%** | **50-75%** | **75-100%** |
| --- | --- | --- | --- | --- |
| <3 months |  |  |  |  |
| 3-6 months |  |  |  |  |
| 6 - 12 months |  |  |  |  |
| 12 years |  |  |  |  |
| 25 years |  |  |  |  |
| > 5 years |  |  |  |  |

1. **In dogs diagnosed and/or treated, indicate the % of euthanasia performed:**  ___________________

15. **What are reasons for or situations in which you would consider euthanasia?**

|  | **Owner request** | **Advice of vet** |
| --- | --- | --- |
| Young children in family |  |  |
| Elderly in family |  |  |
| Immunosuppressed individuals in family |  |  |
| Other dogs in the house |  |  |
| Dog shelters |  |  |
| Cost of treatment |  |  |
| Treatment intolerance |  |  |
| Renal disease |  |  |
| Other severe diseases |  |  |
| Other (specify): |  |  |

1. **Indicate which of the following preventive measures recommended in your veterinary clinic for the control of canine leishmaniosis**

- Annual serological check-ups
- Use of repellents
- Vaccination against leishmaniosis
- Keeping dogs indoors from dusk to dawn
- Use of mosquito nets
- Other (specify):

1. **Which repellents/insecticides do you recommend for the control of CanL?**

|  | Never | Sometimes Often Always | Often | Always |
| --- | --- | --- | --- | --- |
| Advantix (Bayer) |  |  |  |  |
| Effitix (Virbac) |  |  |  |  |
| Exspot (MSD) |  |  |  |  |
| Frontline Tri-Act (Merial) |  |  |  |  |
| Scalibor (MSD) |  |  |  |  |
| Seresto (Bayer) |  |  |  |  |
| Vectra 3D (Ceva) |  |  |  |  |
| Other (specify): |  |  |  |  |

1. **Indicate the frequency of vaccination against CanL at your veterinary clinic**

|  | Never | Sometimes Often Always | Often | Always |
| --- | --- | --- | --- | --- |
| Canileish® (Virbac) |  |  |  |  |
| Letifend® (Leti) |  |  |  |  |

1. **Do you perform any serological test prior to vaccination?**

- Yes, quantitative serology (IFAT or ELISA)
- Yes, rapid test
- No

1. **Do you perform any serological test prior to re-vaccination?**

- Yes, quantitative serology (IFAT or ELISA)
- Yes, rapid test
- No

1. **Do you recommend the use of repellents in vaccinated animals?**

- Never
- Sometimes
- Always

1. **Indicate the NUMBER of vaccinated animals that have developed clinical leishmaniosis (over a period of 5 years) after annual vaccination**  ______________
2. **In the case of having diagnosed a dog with clinical leishmaniosis once vaccinated, how was the diagnosis made?**

- Qualitative serology (rapid test)
- Quantitative serology (IFAT and/or ELISA)
- Alteration in haematology, biochemistry, electrophoretogram, urinalysis
- Presence of clinical signs compatible with the disease
- Bone marrow and/or lymph node PCR
- Other (specify):

**23. Indicate what adverse effects associated with vaccination you have observed**

|  | Never | Sometimes Often Always | Often | Always |
| --- | --- | --- | --- | --- |
| Pain and inflammation at the point of inoculation |  |  |  |  |
| Erythema at the point of inoculation |  |  |  |  |
| Hyperthermia and apathy |  |  |  |  |
| Vomiting and diarrhoea |  |  |  |  |
| Hypersensitivity reactions |  |  |  |  |
| Other (specify): |  |  |  |  |

24. **Have you diagnosed cases of feline leishmaniosis?___Yes ___No. If yes, indicate the number and clinical signs observed __________________________________**

25. **Do you explain to dog owners the zoonotic nature of this disease?**

- Never
- Rarely
- Sometimes
- Often
- Always

26. **Have you heard of the scientific association LeishVet?**

- Yes
- No

**27. How did you hear for the first time about LeishVet?**

- In a talk or presentation on vector diseases
- Via a laboratory
- Via your University
- Via a journal
- Via the internet
- Via a colleague
- Via this survey

28. **Have you used the LeishVet guidelines to gain information about canine and feline leishmaniosis?**

- Yes
- No
